# Liver-specific Bcl3 Knockout Alleviates Acetaminophen-induced Liver Injury by Activating Nrf2 Pathway in Male Mice

**DOI:** 10.1016/j.jcmgh.2025.101483

**Published:** 2025-02-25

**Authors:** Jingtao Gao, Wei Lu, Yue Xin, Haowen Ma, Xiaohang Sheng, Ge Gao, Xue Kang, Shan Jiang, Yuxin Zhao, Yang Lv, Yuna Niu, Yinming Liang, Hui Wang

**Affiliations:** 1 Department of Immunology, Basic Medical College of Xinjiang Medical University, Urumqi, Xinjiang, China; 2 Henan Key Laboratory of Immunology and Targeted Drugs, Xinxiang Medical University, Xinxiang, Henan, China; 3 Cardiac Center, Beijing Luhe Hospital Capital Medical University, Tongzhou, Beijing, China; 4 Henan Collaborative Innovation Center of Molecular Diagnosis and Laboratory Medicine, School of Medical Technology, Xinxiang Medical University, Xinxiang, Henan, China; 5 Laboratory of Genetic Regulators in the Immune System, School of Medical Technology, Xinxiang Medical University, Xinxiang, Henan, China

**Keywords:** Antioxidant, APAP overdose, GSH Synthesis, Hepatotoxicity, Oxidative Stress

## Abstract

**Background & Aims:**

Acetaminophen (APAP) overdose is the leading cause of acute liver failure, with oxidative stress being a critical factor in this process. Glutathione (GSH) plays a vital defensive role. Activation of nuclear factor erythroid 2 like 2 (Nrf2) pathway mitigates APAP-induced liver damage by promoting GSH biosynthesis and enhancing drug detoxification. Although the role of B cell leukemia/lymphoma 3 (Bcl3) in regulating inflammatory responses, cellular oncogenesis, and immune balance is well-documented, its function in APAP-induced liver injury remains unclear.

**Methods:**

We employed liver-specific Bcl3 knockout (*Bcl3*^*hep-/-*^) mice and adeno-associated virus (AAV)-8-mediated Bcl3 overexpression (AAV-Bcl3) mice to model APAP-induced liver injury. Liver damage was assessed through hematoxylin and eosin staining and serum alanine aminotransferase and aspartate aminotransferase measurements. The interaction between Bcl3 and Nrf2 was examined using immunofluorescence and co-immunoprecipitation assays.

**Results:**

Our study reveals a significant upregulation of Bcl3 expression in the livers of male mice following APAP administration, suggesting Bcl3’s potential involvement in this pathological process. In *Bcl3*^*hep-/-*^ mice, a reduced severity of liver damage was observed at both 6 and 24 hours post-APAP treatment compared with controls. Notably, Bcl3-deficient mice exhibited accelerated GSH replenishment due to the rapid induction of *Gclc* and *Gclm* genes following 6 hours of APAP exposure. Through immunofluorescence and co-immunoprecipitation analyses, we identified an interaction between Bcl3 and Nrf2. The loss of Bcl3 enhanced Nrf2 translocation upon APAP challenge, leading to the upregulation of antioxidant gene expression. These findings suggest that Bcl3 knockout alleviates oxidative stress resulting from APAP overdose.

**Conclusion:**

We uncovered a previously uncharacterized role of Bcl3 in APAP-induced liver injury, emphasizing the role of the Bcl3-Nrf2 axis in oxidative stress-related liver damage.

## Graphical Abstract

**Figure:**
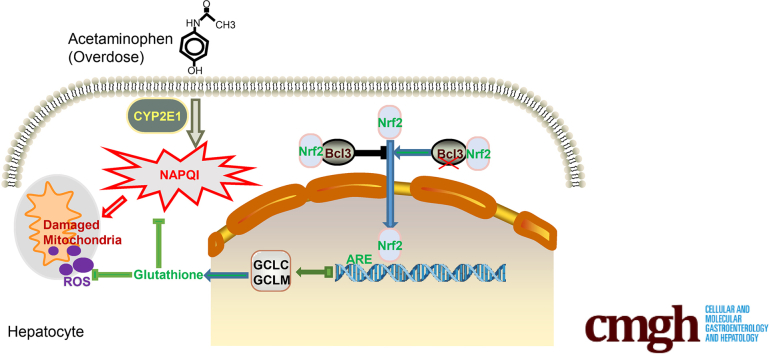

**Summary**

This study investigates the role of B cell leukemia/lymphoma 3 (Bcl3) in acetaminophen (APAP)-induced liver injury, revealing that Bcl3 deficiency reduces liver damage by enhancing nuclear factor erythroid 2 like 2 (Nrf2)-mediated antioxidant responses. These findings highlight a novel Bcl3-Nrf2 interaction in mitigating oxidative stress during APAP overdose.

Acetaminophen (paracetamol) (APAP) is the most widely used pain reliever globally.^1^ Despite its efficacy in pain relief, APAP overdose is the leading cause of acute liver failure in the United States and many Western nations.^2^ The hepatic metabolism of APAP primarily occurs through Phase II enzymes, specifically UDP-glucuronosyltransferase and sulfotransferase, which facilitate its conversion for elimination. A minor fraction is metabolized by cytochrome P450 enzymes, such as CYP2E1, CYP3A4, and CYP1A2, forming the hepatotoxic metabolite N-acetyl-p-benzoquinone imine (NAPQI). NAPQI is known to deplete glutathione levels and subsequently induce oxidative stress and mitochondrial dysfunction through reactive oxygen species (ROS) generation.^3^^,^^4^ This mitochondrial damage is further exacerbated by the activation of the mitogen-activated protein kinase (MAPK) signaling cascade, with continued downstream C-jun N-terminal kinase (JNK) signaling aggravating oxidative damage, ultimately leading to hepatocyte necrosis.^5^, ^6^, ^7^ The complexity of APAP-induced liver injury extends beyond these pathways, with additional factors such as endoplasmic reticulum stress, nitrotyrosine accumulation, and mitochondrial dysfunction contributing to the hepatic pathology.^8^ N-acetyl cysteine (NAC), which promotes the re-synthesis of glutathione (GSH), is currently the only antidote approved by the United States Food and Drug Administration (FDA) for treating APAP-induced liver injury. However, concerns remain about its limited efficacy and potential side effects with prolonged treatment.^9^

B cell leukemia/lymphoma 3 (Bcl3), an atypical Ikappa B (IkB) family member, is a pivotal modulator of nuclear factor kappa B (NF-κB) signaling, known for its profound influence on inflammatory responses and its diverse role in immunity.^10^^,^^11^ Initially recognized as a proto-oncogene in B cell leukemia, Bcl3 has since been disclosed to serve a dual function as both an inhibitor and a coactivator of NF-κB p50 and p52 subunits, underscoring its multifaceted nature.^12^ Apart from its canonic role in inflammation responses,^13^, ^14^, ^15^, ^16^ Bcl3 has also a distinct function in immunomodulation,^11^^,^^17^, ^18^, ^19^, ^20^ cellular development,^21^, ^22^, ^23^, ^24^ and metabolic pathways.^25^, ^26^, ^27^ It is intriguing that Bcl3 exhibits either pro-inflammatory or anti-inflammatory roles in different microenvironments.^28^

Recent research has begun to unravel Bcl3’s involvement in hepatic function and disease. A 2016 study identified Bcl3 as a promoter of hepatocellular carcinoma growth through the modulation of cyclin D1.^29^ In the same year, Gehrke et al found that Bcl3 promotes hepatic steatosis through modulation of peroxisome proliferator-activated receptor alpha (PPARα) and peroxisome proliferator-activated receptor-gamma coactivator (PGC-1α).^30^ For liver regeneration, Bcl3 forms a complex with yes-associated protein 1 (YAP1) to promote Sox9+HNF4α+ hepatocytes to participate in liver regeneration.^31^ Bcl3 exhibits distinct functions in a different model of liver injury; it provides protection against D-galactosamine (d-GalN)/lipopolysaccharide (LPS) and FAS-ligand-triggered hepatocyte apoptosis^32^; conversely, it appears to aid in tumor necrosis factor (TNF)-induced hepatocyte apoptosis by mediating receptor interacting protein (RIP1) deubiquitination.^33^ However, the role of Bcl3 in oxidative stress-induced hepatocyte necrosis has remained unresolved. In our study, a well-established acute liver injury model was employed to investigate the influence of Bcl3 on APAP-induced hepatocytes necrosis. We found that Bcl3 deficiency confers significant protection against APAP-induced hepatotoxicity in both murine models and isolated hepatocytes. This protection was achieved through enhanced translocation of nuclear factor erythroid 2 like 2 (Nrf2), which promotes the synthesis of GSH, thereby mitigating oxidative stress-induced necrosis. Taken together, our results reveal that Bcl3-Nrf2 axis plays a critical role in APAP-induced acute liver injury.

## Results

### The Expression of Bcl3 is Significantly Increased in Liver Tissue and Primary Hepatocytes of Mouse With APAP Treatment

**Figure 1:**
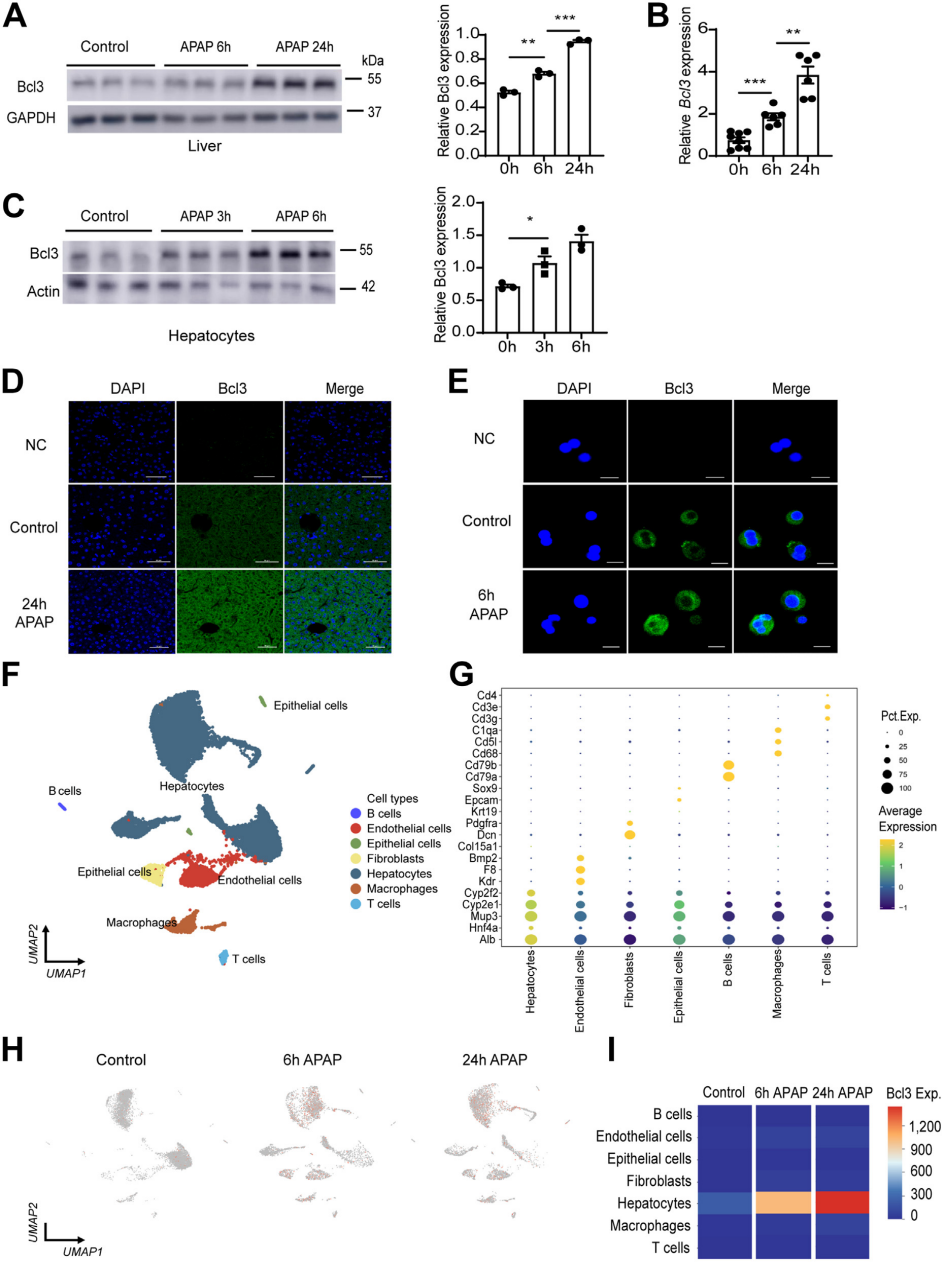
**Elevated expression of Bcl3 both in liver and primary hepatocytes of mice with APAP treatment.** (*A*) Western blot analysis and quantification of Bcl3 expression in liver tissues from mice treated with APAP for 0 and 24 hours. (*B*) Quantification of *Bcl3* expression in liver tissues via qPCR (n = 6 per group). (*C*) Western blot analysis and quantification of Bcl3 expression in primary hepatocytes from mice treated with APAP (10 mM) for 0, 3, and 6 hours. (*D and E*) Immunofluorescence staining of Bcl3 in liver tissues (scale bar, 50 μm) and primary hepatocytes (scale bar, 10 μm) of mice following APAP treatment. (*F*) Analysis of a publicly available snRNA-seq dataset (GEO accession number: GSE223558) from liver tissue of both saline- and APAP-treated mice, using an unsupervised clustering approach with Uniform Manifold Approximation and Projection (UMAP) visualization. Cells are colored according to their cell type annotations. (*G*) Marker genes of the seven cell clusters. Pct. Exp. means percent expression, referring to the percentage of cells in a population that express a particular gene. Average Expression is calculated using a log2 transformation. (*H*) The expression of Bcl3 in mice treated with APAP for 0, 6, and 24 hours are shown in UMAP plot. (*I*) Quantification of Bcl3 expression across 7 clusters of cells. Data represent 3 independent experiments. Data are presented as mean ± SEM. Student’s *t* test (A, B, C), ∗*P* < .05; ∗∗*P* < .01; ∗∗∗*P* < .001.

To investigate whether Bcl3 plays a role in APAP-induced liver injury, we measured the hepatic expression of Bcl3 in a mouse model of APAP-induced liver injury. Western blotting and quantitative polymerase chain reaction (qPCR) experiments demonstrated that a toxic dose of APAP (300 mg/kg) significantly increased Bcl3 expression in the livers of treated mice compared with control mice (Figure 1*A and B*). Consistent with these results, Bcl3 expression was also significantly elevated in primary hepatocytes isolated from the livers of mice treated with 10 mM APAP for 3 hours and 6 hours (Figure 1*C*). Additionally, immunofluorescence staining revealed a marked increase in Bcl3 expression in both liver tissue (Figure 1*D*) and primary hepatocytes (Figure 1*E*) following APAP treatment. To further investigate the changes in Bcl3 expression following APAP treatment, we analyzed a publicly available single-nucleus RNA sequencing (snRNA-seq) dataset (GSE223558) derived from liver tissue of both saline- and APAP-treated mice. After filtering out low-quality cells and classifying based on gene expression patterns, we identified a total of 7 distinct cell clusters (Figure 1*D*). Using this snRNA-seq data, we compared the transcriptome expression of Bcl3 in the mouse liver. Bcl3 was found to be highly expressed in hepatocytes and was significantly upregulated following 6 and 24 hours of APAP treatment. (Figure 1*H and I*). The marker genes for the 7 cell clusters are presented in Figure 1*G*. These results suggest that APAP treatment enhances Bcl3 expression, indicating a potential role for Bcl3 in APAP-induced liver injury.

### Liver-specific Knockout of Bcl3 Protects Mice From APAP-induced Liver Injury

**Figure 2:**
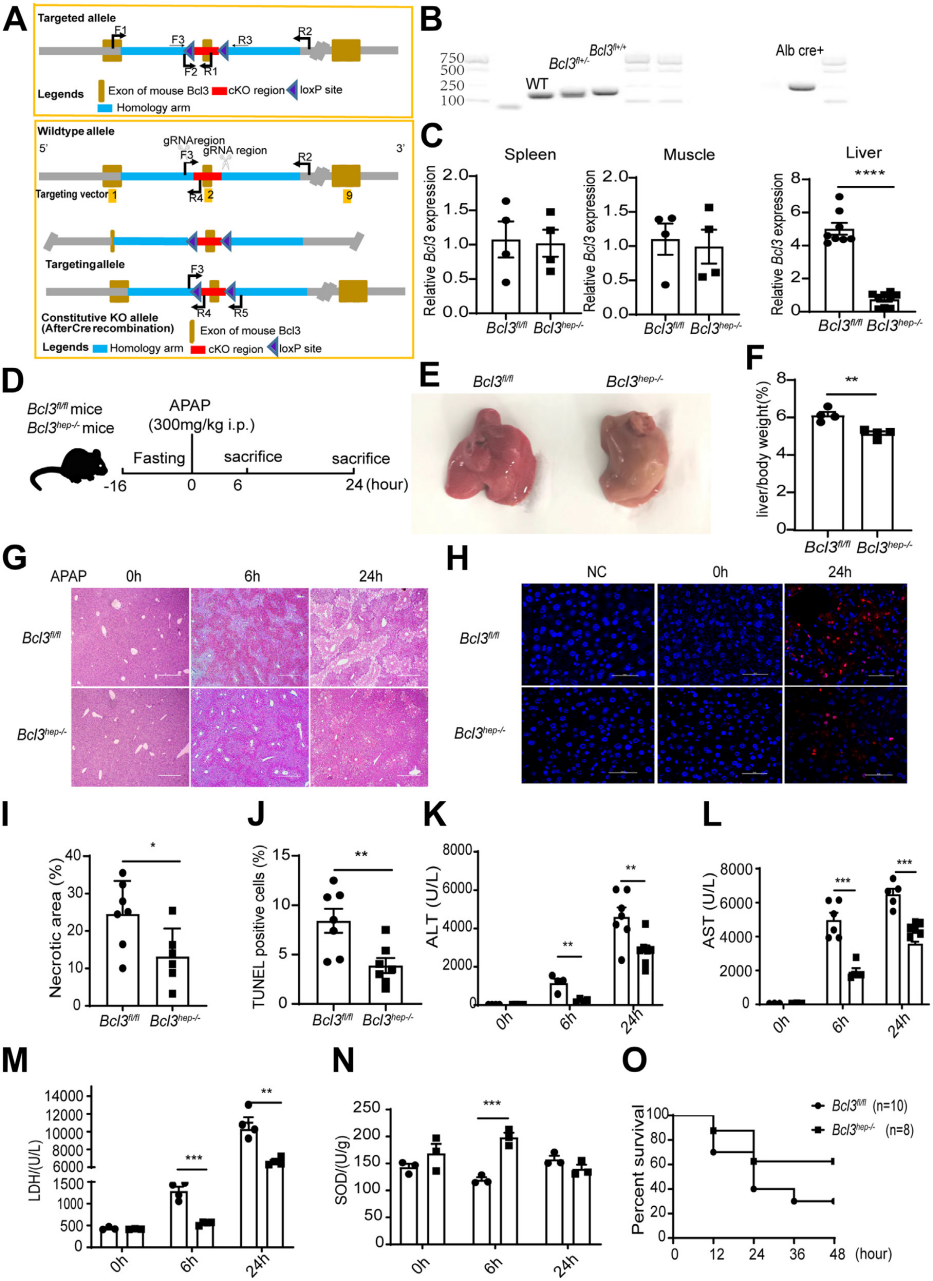
**Liver-specific knockout of Bcl3 protects mice from APAP-induced acute liver injury.** (*A*) Design of *Bcl3*^*flox/flox*^ mice. (*B*) Genotyping of *Bcl3*^*flox/flox*^ and Albumin-cre using PCR. (*C*) Relative Bcl3 expression in the liver, spleen, and muscle of *Bcl3*^*fl/fl*^ and *Bcl3*^*hep-/-*^ mice, assessed by qPCR. (*D*) APAP treatment scheme. Male *Bcl3*^*fl/fl*^ and *Bcl3*^*hep-/-*^ mice were fasted for 16 hours, injected intraperitoneally with APAP (300 mg/kg), and sacrificed at 0, 6, and 24 hours post-injection. (*E*) Representative liver morphologies of *Bcl3*^*fl/fl*^ and *Bcl3*^*hep-/-*^ mice at 6 hours post-APAP treatment. (*F*) Quantification of liver weight normalized to body weight in mice treated with APAP for 24 hours (n = 4 per group). (*G, I*) H&E staining of liver sections (scale bar, 500 μm) and quantification of necrotic areas in liver tissues following APAP treatment for 24 hours (n = 7). (*H*) TUNEL staining in liver tissues and quantification of TUNEL-positive cell rates. (*K–M*) Serum levels of ALT, AST, and LDH in mice (n = 4–7 per group). (*N*) Quantification of SOD in liver tissues (n = 3). Data represent 3 independent experiments. (*O*) Survival curve of *Bcl3*^*fl/fl*^ (n = 10) and *Bcl3*^*hep-/-*^ mice (n = 8) following a lethal dose of APAP (750 mg/kg, intraperitoneally). Data are presented as mean ± SEM. Student’s *t* test, ∗∗*P* < .01; ∗∗∗*P* < .001.

To explore the function of Bcl3 in the liver, the *Bcl3*^*fl/fl*^ mice were designed as in Figure 2*A*, and liver-specific Bcl3 knockout (*Bcl3*^*hep-/-*^) mice were generated by crossbreeding the *Bcl3*^*fl/fl*^ mice with the liver-specific Albumin-Cre transgenic mice. The *Bcl3*^*fl/fl*^ and *Bcl3*^*hep-/-*^ mice were genotyped by PCR (Figure 2*B*), and the genotyping primers for mouse tail identification are shown in Table 1. The efficiency of Bcl3 knockout in the liver, but not in the muscle and spleen, were verified by qPCR (Figure 2*C*). For the liver injury model, *Bcl3*^*hep-/-*^ mice and *Bcl3*^*fl/fl*^ littermates were fasted overnight (16 hours), and tissues were harvested 6 and 24 hours after APAP administration (Figure 2*D*). *Bcl3*^*hep-/-*^ mice exhibited less liver congestion upon visual inspection of intact organs after 6 hours of APAP treatment (Figure 2*E*). After 24 hours of APAP treatment, *Bcl3*^*hep-/-*^ mice manifested a lower liver-to-body weight ratio (Figure 2*F*). Notably, histologic analysis showed significantly less hepatocyte necrosis in *Bcl3*^*hep-/-*^ mice compared with *Bcl3*^*fl/fl*^ mice as evidenced by hematoxylin and eosin (H&E) staining and terminal deoxynucleotidyl transferase dUTP nick end labeling (TUNEL) staining (Figures 2*G–J*). Additionally, the APAP-induced elevations of circulating alanine aminotransferase (ALT) and aspartate aminotransferase (AST) levels were attenuated in *Bcl3*^*hep-/-*^ mice at 6 and 24 hours, as well lactate dehydrogenase (LDH) levels (Figure 2*K–M*). Conversely, levels of superoxide dismutase (SOD) were higher in the liver of *Bcl3*^*hep-/-*^ mice after APAP treatment for 6 hours (Figure 2*N*). Furthermore, *Bcl3*^*hep-/-*^ mice showed an observably improved survival rate compared with *Bcl3*^*fl/fl*^ mice when treated with a lethal dose (750 mg/kg) of APAP (Figure 2*O*). These findings suggest that Bcl3 deficiency alleviates APAP-induced acute liver injury.

**Table 1:** Primers Used for PCR Analysis

Primer name	Sequence (5′ to 3′)	Function
Bcl3flox/flox-F	TTATATGGGTGCTTGTCCCTGTG	Genotyping of Bcl3flox mice
Bcl3flox/flox-R	CAAGTTAGGGCCACATAGTGAGATC	
Albmin-cre-F	AGCGATGGATTTCCGTCTCTGG	Genotyping of Alb-cre mice
Albmin-cre-R	AGCTTGCATGATCTCCGGTATTGAA	

PCR, polymerase chain reaction.

### Bcl3 Knockout Promotes GSH Biosynthesis Through Nrf2 Signaling During the Early Phase of APAP Metabolism

**Figure 3:**
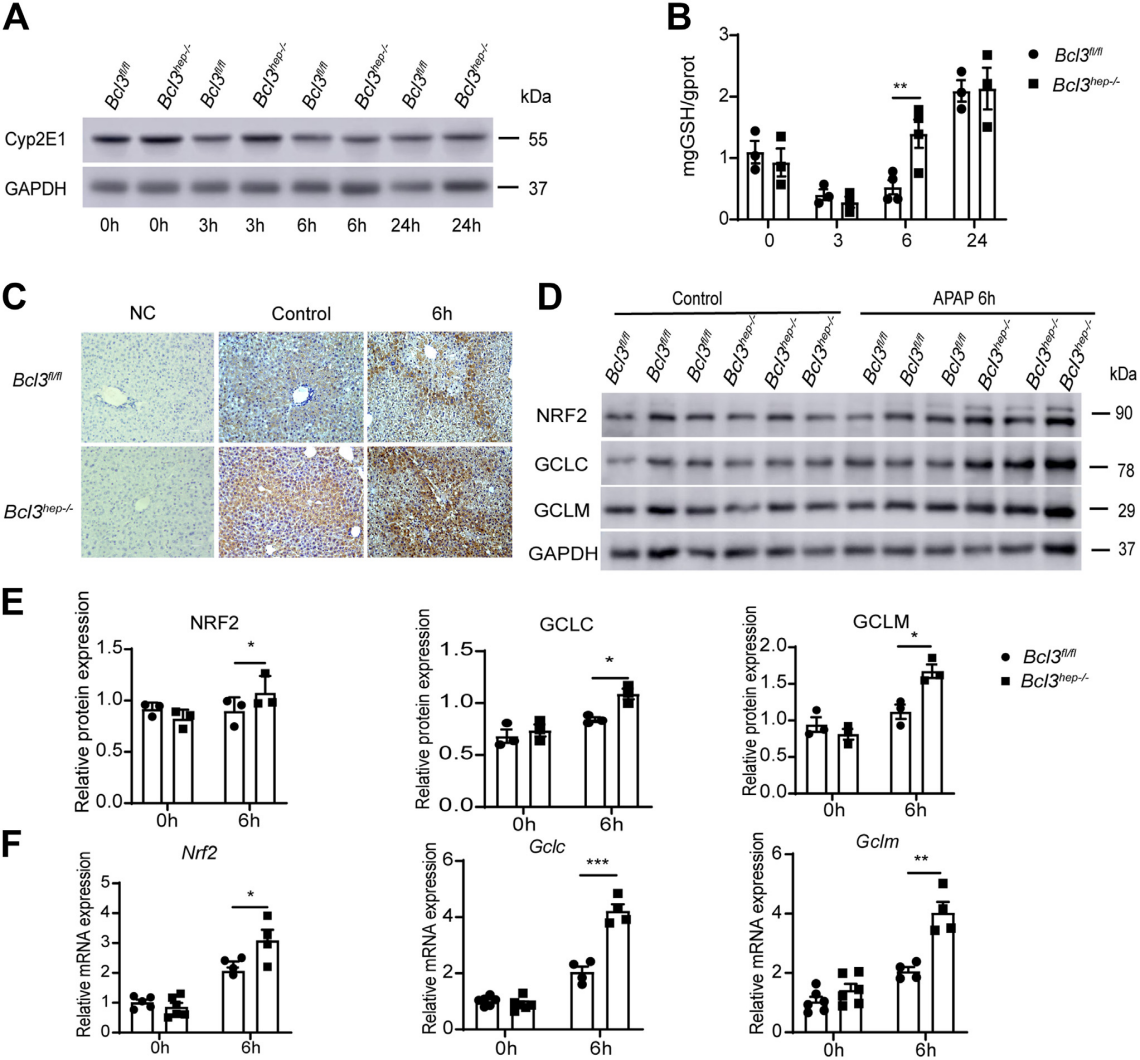
**Bcl3 deficiency enhances GSH recovery through Nrf2 signaling.** (*A, B*) *Bcl3*^*fl/fl*^ and *Bcl3*^*hep-/-*^ mice were treated with APAP (300 mg/kg, intraperitoneally), and liver tissues were collected at 0, 3, 6, and 24 hours post-treatment. (*A*) Western blot analysis of Cyp2E1 levels in liver tissues. (*B*) Quantification of GSH levels in liver tissues using a GSH assay kit (n = 3–5 per group). (*C*) Immunohistochemical staining of GCLC in the livers of *Bcl3*^*fl/fl*^ and *Bcl3*^*hep-/-*^ mice treated with APAP (300 mg/kg, intraperitoneally) for 0 and 6 hours. (*D*) Western blot analysis of Nrf2, GCLC, and GCLM protein levels in liver tissues. (*E*) Quantification of protein levels. (*F*) qPCR analysis of *Nrf2*, *Gclc,* and *Gclm* mRNA levels in liver tissues (n = 4–6 per group). Data represent 3 independent experiments and are presented as mean ± SEM. Statistical significance was determined by Student’s t-test (*B, E, F*); ∗*P* < .05; ∗∗*P* < .01; ∗∗∗*P* < .001.

Cytochrome P450 2E1 (Cyp2E1) plays a vital role in the early phase of APAP metabolism, generating toxic metabolite NAPQI in hepatocytes. Reactive NAPQI depletes intracellular GSH and covalently binds to mitochondrial proteins when GSH is exhausted.^34^ Therefore, we detected the protein level of Cyp2E1 and the contents of GSH in the liver at 0, 3, 6, and 24 hours after APAP treatment. However, it was found that Bcl3 deficiency in hepatocytes did not affect the expression of Cyp2E1 (Figure 3*A*). Interestingly, the level of GSH was found to be higher in the liver of *Bcl3*^*hep-/-*^ mice at 6 hours after APAP treatment (Figure 3*B*). Next, we explored whether loss of Bcl3 improved the biosynthesis of GSH, thus contributing to reduced liver injury. Glutamate-cysteine ligase catalytic subunit (GCLC) is the first rate-limiting enzyme in GSH synthesis. Immunochemistry showed higher expression of GCLC in the liver of *Bcl3*^*hep-/-*^ mice (Figure 3*C*). Nrf2, a transcription factor, plays a vital role in antioxidant stress through the regulation of downstream target GCLC and glutamate-cysteine ligase modifier subunit (GCLM). Impressively, *Bcl3*^*hep-/-*^ mice exhibited higher expression of both NRF2 and GCLM at the protein and mRNA levels (Figure 3*D–F*).

### Bcl3 Deficiency Mitigates APAP-induced Primary Hepatocyte Death

**Figure 4:**
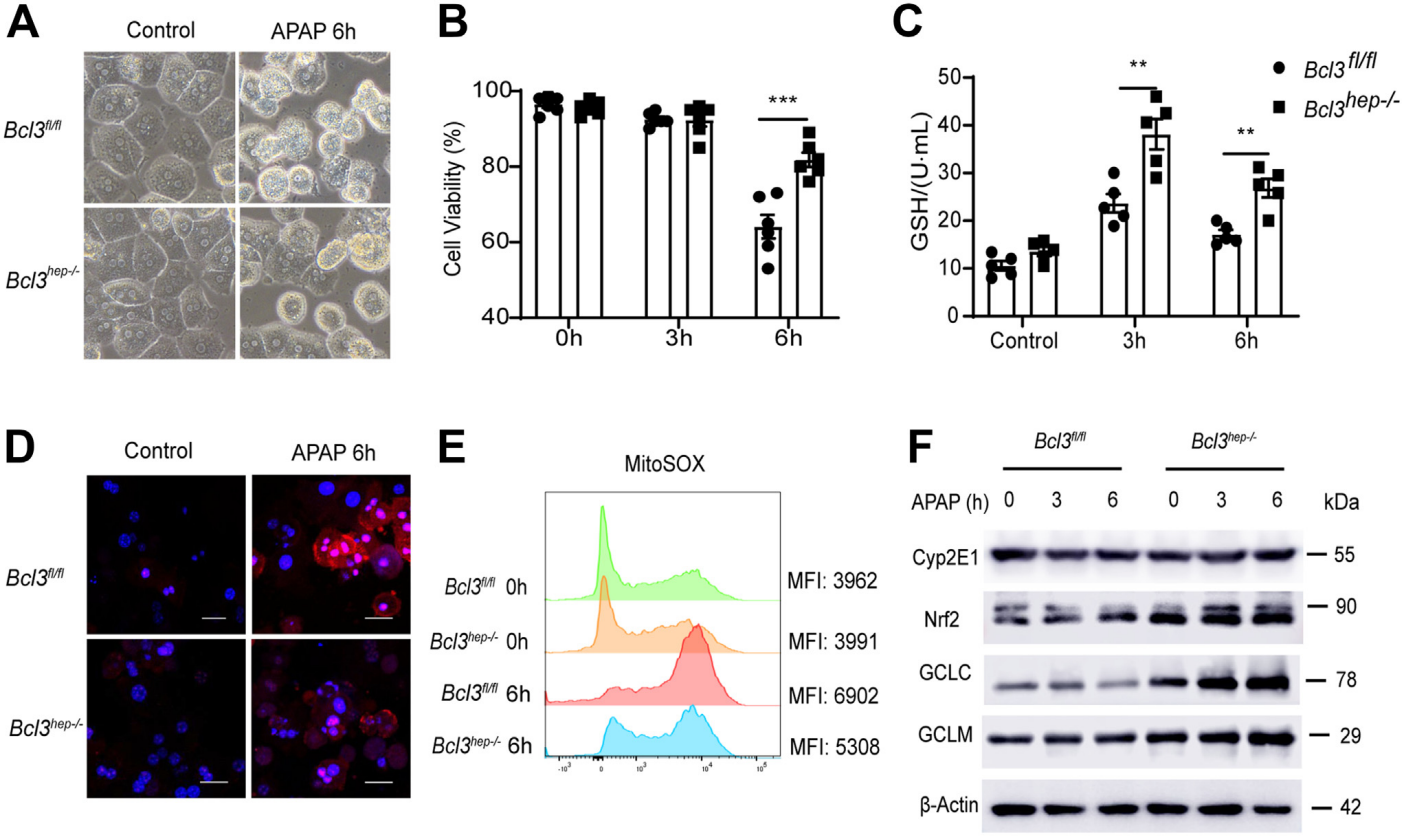
**Bcl3 deficiency mitigates APAP-induced primary hepatocyte death.** (*A*) Primary hepatocytes were isolated from *Bcl3*^*hep-/-*^ mice and *Bcl3*^*fl/fl*^ mice. Representative images of primary hepatocytes treated with APAP (10 mM) for 0 and 6 hours. (*B*) Cell viability of primary hepatocytes measured using the CCK8 assay. (*C*) GSH levels in primary hepatocytes after APAP treatment for 3 and 6 hours. (*D*) MitoSOX staining in primary hepatocytes detected by immunofluorescence (scale bar, 10 μm). (*E*) Quantification of MitoSOX mean fluorescence intensity in primary hepatocytes by flow cytometry. (*F*) Protein levels of Cyp2E1, Nrf2, GCLC, and GCLM in primary hepatocytes treated with APAP for 3 and 6 hours, as determined by Western blotting. Data represent 3 independent experiments. Data are presented as mean ± SEM. Student’s *t*-test (*B, C*), ∗∗*P* < .01; ∗∗∗*P* < .001.

Primary hepatocytes from the *Bcl3*^*hep-/-*^ mice and *Bcl3*^*fl/fl*^ mice were used to confirm the protective function of Bcl3 against APAP-induced hepatotoxicity. Primary hepatocytes from *Bcl3*^*fl/fl*^ exposed to APAP (10 mM) for 6 hours showed clear morphologic changes with some nuclei not even observable. In contrast, Bcl3-knockout hepatocytes still relatively maintained structural integrity (Figure 4*A*), as evidenced by cell counting kit-8 (CCK8) assay (Figure 4*B*). The GSH level in Bcl3-knockout hepatocytes was significantly lower than that in the control group after 3 hours and 6 hours of APAP treatment (Figure 4*C*). Excessive APAP metabolism leads to the formation of NAPQI, which binds to mitochondrial proteins, causing dysfunction and the subsequent production of large amounts of ROS. Mitochondrial superoxide levels were measured using MitoSOX staining. Consistent with the above results, the mitochondrial accumulation of superoxide was markedly decreased after APAP treatment in the absence of Bcl3 (Figure 4*D and E*). Additionally, although the protein expression of Cyp2E1 and Nrf2 showed no significant differences, the expression of GCLC and GCLM was higher in Bcl3-knockout hepatocytes compared with controls (Figure 4*F*).

### AAV-mediated Bcl3 Overexpression in Mice Liver Aggravates APAP-induced Liver Injury

**Figure 5:**
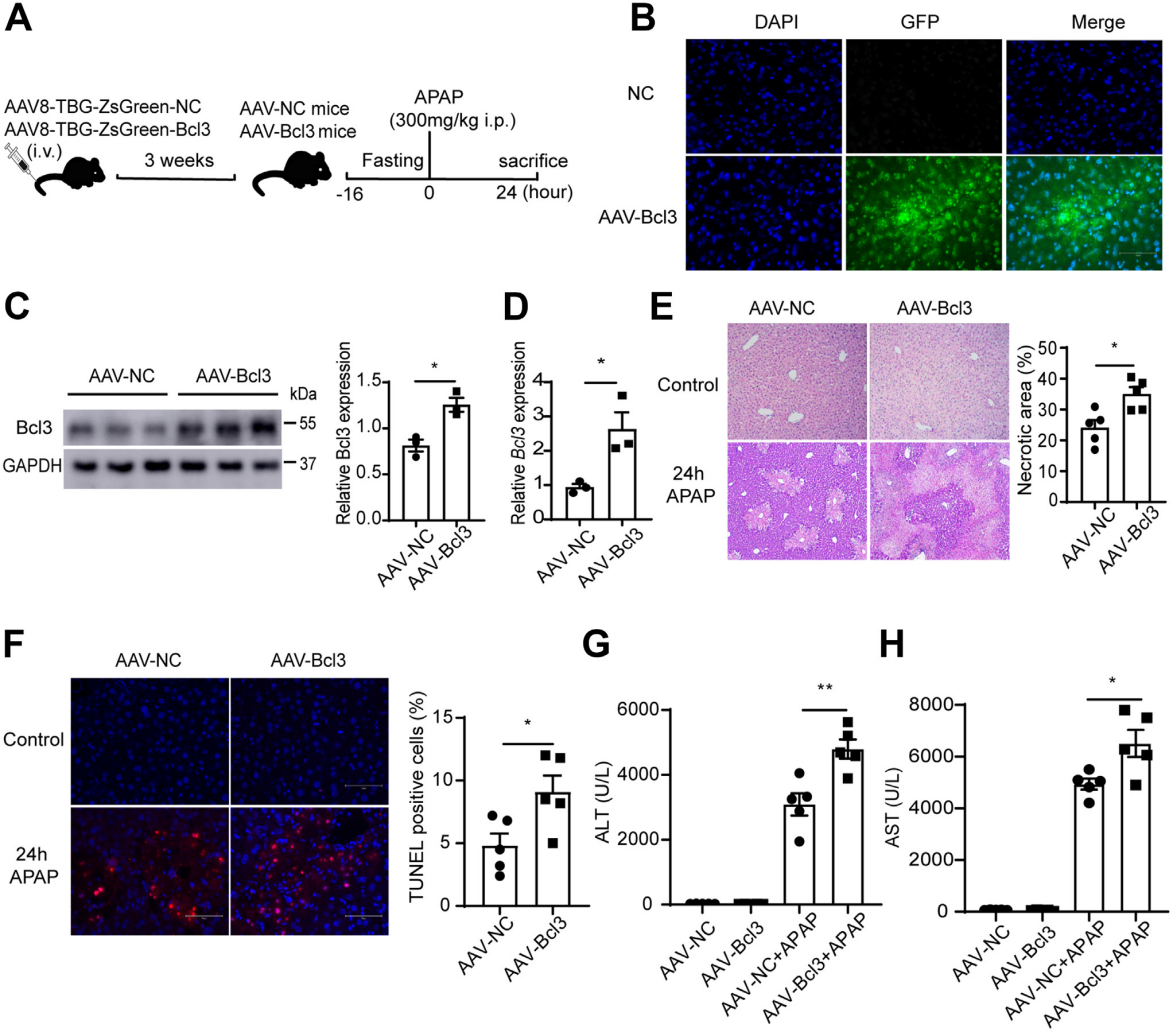
**AAV8-meadiated Bcl3 overexpression exacerbates APAP-induced liver injury.** (*A*) Experimental scheme: wild-type mice were injected intravenously with AAV8-TBG-ZsGreen-Bcl3 for 3 weeks to induce Bcl3 overexpression in the liver. Following a 16-hour fast, the mice were administered APAP (300 mg/kg, intraperitoneally) and were sacrificed 24 hours later for tissue collection. (*B*) Detection of the AAV8-TBG-m-Bcl3-3×flag-ZsGreen construct overexpressing Bcl3 in mouse liver by fluorescence microscopy. (*C*) Western blot analysis of Bcl3 expression after 3 weeks of AAV treatment. (*D*) Relative Bcl3 expression in the liver of AAV-NC and AAV-Bcl3 mice, determined by qPCR (n = 3). (*E and F*) Representative images of H&E and TUNEL staining and quantification of TUNEL-positive cells in liver sections from mice in (*A*) after 24 hours of APAP treatment (n = 5 per group). (*G and H*) Serum ALT and AST levels. Data represent 3 independent experiments and are presented as mean ± SEM. Statistical analysis was performed using Student *t*-test, ∗*P* < .05; ∗∗*P* < .01.

To further investigate whether overexpression of Bcl3 exacerbates APAP-induced hepatotoxicity *in vivo*, mice were injected with AAV8 vectors containing either a control or Bcl3 gene and subsequently treated with APAP for 24 hours (Figure 5*A*). The efficiency of Bcl3 overexpression was measured by fluorescence microscopy (Figure 5*B*), Western blotting (Figure 5*C*), and qPCR experiments (Figure 5*D*). H&E staining revealed that Bcl3 overexpression significantly increased hepatocyte necrosis in the liver (Figure 5*E*). TUNEL staining also showed elevated DNA damage in the liver following Bcl3 overexpression (Figure 5*F*). Furthermore, Bcl3 overexpression markedly increased plasma ALT levels and ALT activity after APAP treatment (Figure 5*E and F*). These data demonstrate that Bcl3 overexpression significantly aggravates APAP-induced liver injury.

### Bcl3 Interacts With the Transcriptional Factor Nrf2

**Figure 6:**
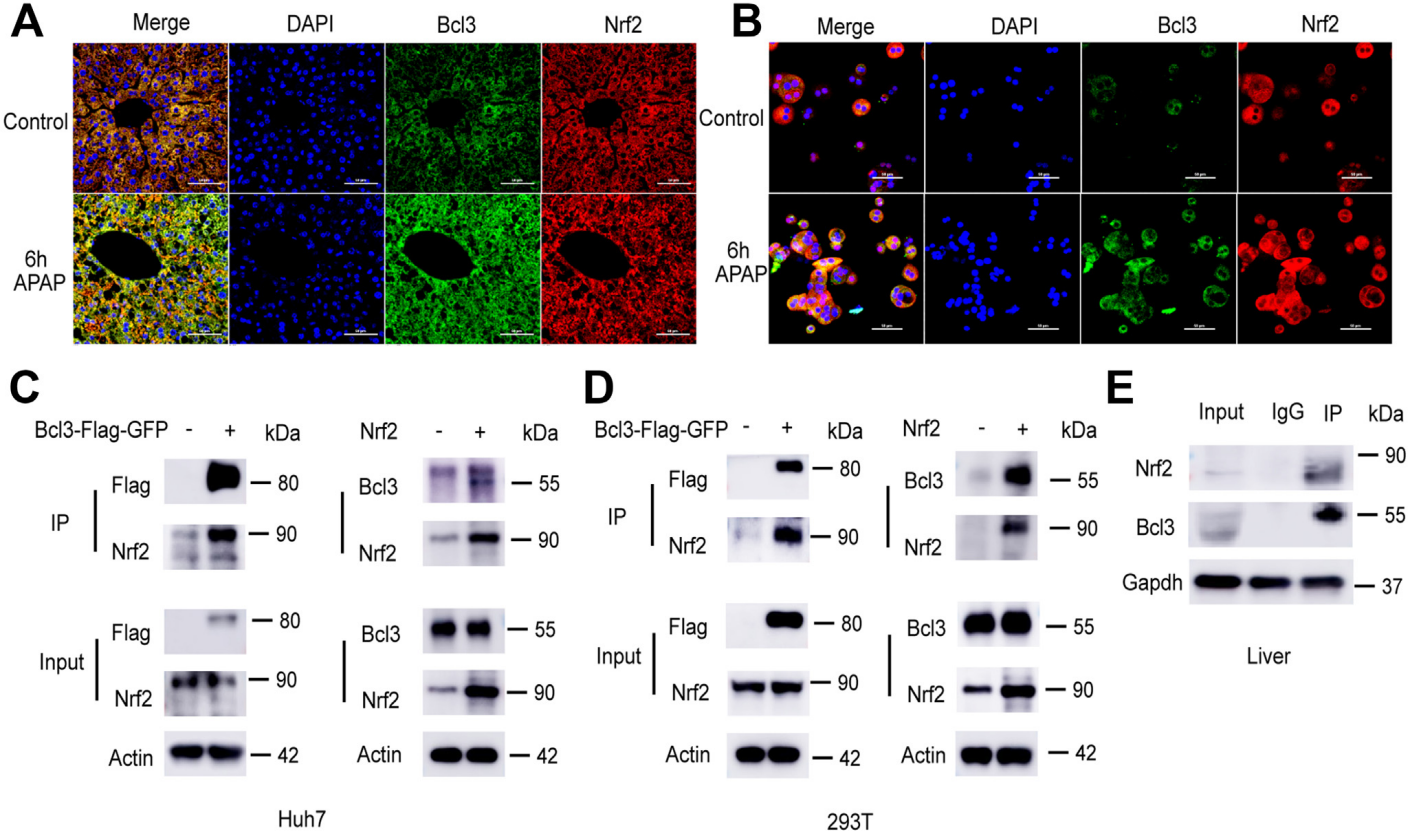
**Bcl3 interacts with Nrf2.** (*A*) Liver tissues from *Bcl3*^*fl/fl*^ and *Bcl3*^*hep-/-*^ mice treated with APAP (300 mg/kg intraperitoneally) for 0 and 6 hours were immunostained with anti-Bcl3 (*green*) and anti-Nrf2 (*red*) antibodies imaged using a confocal microscope (scale bar, 50 μm). (*B*) Primary hepatocytes isolated from *Bcl3*^*hep-/-*^ and *Bcl3*^*fl/fl*^ mice were immunostained for anti-Bcl3 (*green*) and anti-Nrf2 (*red*) following APAP treatment for 0 and 6 hours (scale bar, 10 μm). (*C–D*) Immunoprecipitation analysis of the interaction between Flag-Bcl3 and endogenous Nrf2 as well as Nrf2 and endogenous Bcl3 in Huh7 cells (*C*) and HEK293T cells (*D*) respectively. (*E*) Co-immunoprecipitation analysis using an anti-Bcl3 antibody to pull down Nrf2 protein in liver tissues from WT mice, followed by Western blotting.

**Figure 7:**
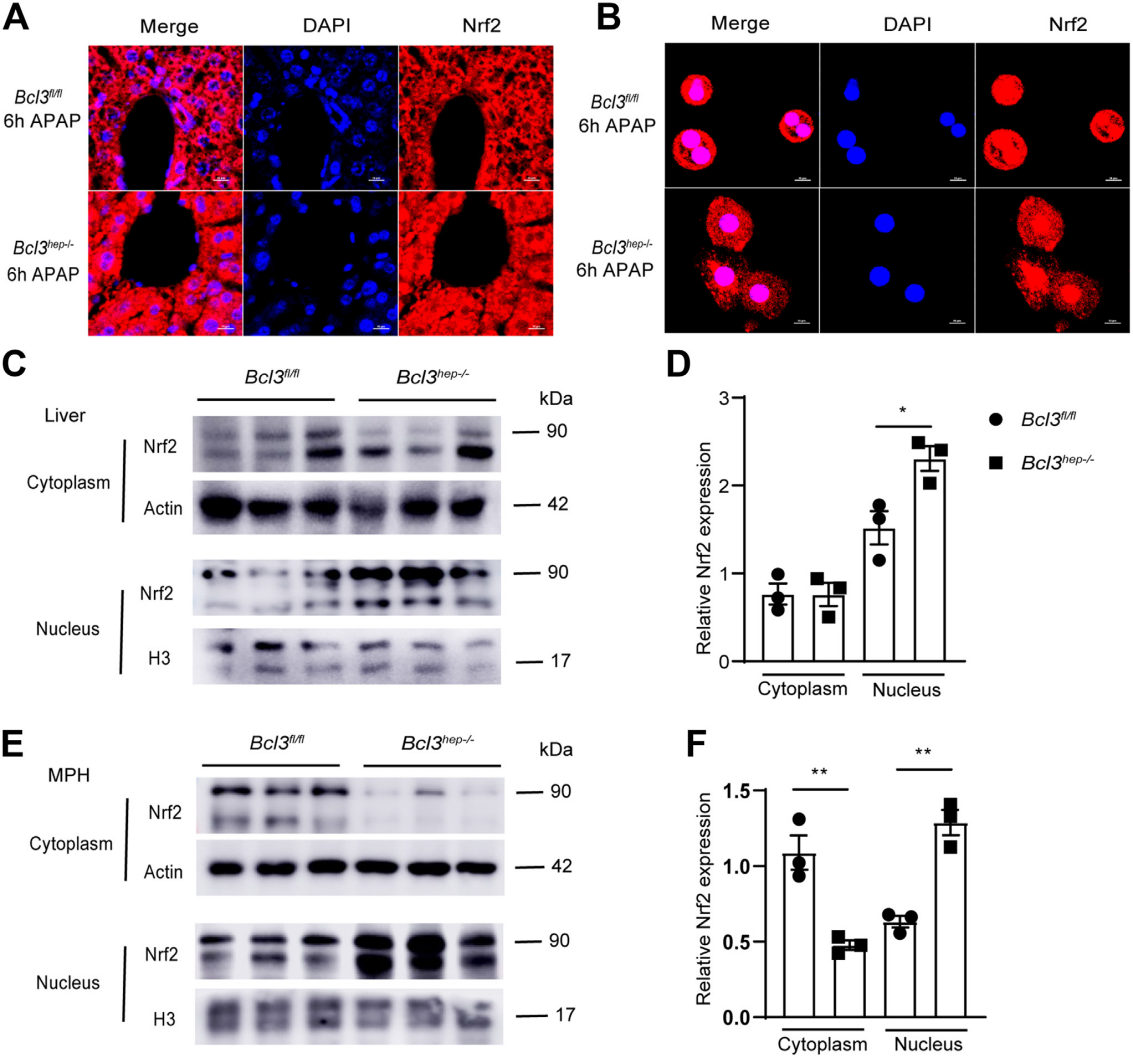
**Bcl3 knockout induces Nrf2 nuclear translocation following APAP treatment.** (*A*) Liver tissues from *Bcl3*^*fl/fl*^ and *Bcl3*^*hep-/-*^ mice treated with APAP (300 mg/kg intraperitoneally) for 6 hours were immunostained for Nrf2 (*red*) (scale bar, 10 μm). (*B*) Primary hepatocytes isolated from *Bcl3*^*hep-/-*^ mice and *Bcl3*^*fl/fl*^ mice were immunostained for Nrf2 (*red*) following APAP treatment for 6 hours (scale bar, 10 μm). (*C*) Liver tissues from *Bcl3*^*fl/fl*^ and *Bcl3*^*hep-/-*^ mice treated with APAP (300 mg/kg intraperitoneally) for 6 hours were subjected to cytoplasmic and nuclear protein extraction. Nrf2 levels in cytoplasmic and nuclear fractions were analyzed by Western blotting (n = 3 per group). (*D*) Quantification of Western blot results. (*E*) Primary hepatocytes were isolated from the *Bcl3*^*hep-/-*^ mice and *Bcl3*^*fl/fl*^ mice. Nrf2 levels in cytoplasmic and nuclear fractions were analyzed by Western blotting (n = 3 per group). (*F*) Quantification of Western blot results. Data represent 3 independent experiments and are presented as mean ± SEM. Student *t*-test (*D, F*), ∗*P* < .05; ∗∗*P* < .01.

The above findings suggested that Bcl3 may interact with Nrf2. Confocal experiments showed that Bcl3 co-localized with Nrf2 both in mouse liver (Figure 6*A*) and primary hepatocytes (Figure 6*B*). Intriguingly, both Bcl3 and Nrf2 were predominantly expressed in the cytoplasm, with APAP treatment increasing their protein levels and promoting their translocation to the nucleus. Moreover, overexpression of Bcl3 or Nrf2 in Huh7 and 293T cells, followed by co-immunoprecipitation, confirmed that Bcl3 interacts with Nrf2 (Figure 6*C and D*). To further validate this interaction, endogenous Bcl3 was immunoprecipitated from mouse liver homogenates, and the presence of Nrf2 in the precipitates was examined. As shown in Figure 6*E*, Bcl3 could bring down Nrf2, and the interaction between them was further validated. Immunofluorescence experiments indicated that Bcl3 deficiency did not affect the expression or spatial distribution of Nrf2 under homeostatic conditions. However, Bcl3 deficiency significantly induced Nrf2 nuclear translocation in the livers of *Bcl3*^*hep-/-*^ mice (Figure 7*A*) and Bcl3-knockout hepatocytes (Figure 7*B*) following APAP treatment compared with controls. Additionally, Western blot analysis of cytoplasmic and nuclear fractions from mouse liver following APAP treatment showed that Bcl3 knockout had no impact on cytoplasmic Nrf2 levels but significantly increased nuclear Nrf2 levels (Figure 7*C and D*). In primary hepatocytes, Bcl3-knockout hepatocytes exhibited lower cytoplasmic and higher nuclear Nrf2 levels after APAP treatment (Figure 7*E and F*). In conclusion, Bcl3 interacts with Nrf2, and Bcl3 deficiency enhances Nrf2 nuclear translocation in response to APAP treatment.

### An Nrf2 Inhibitor (ML385) Counteracted the Protective Effects of Bcl3 on Mice With APAP

**Figure 8:**
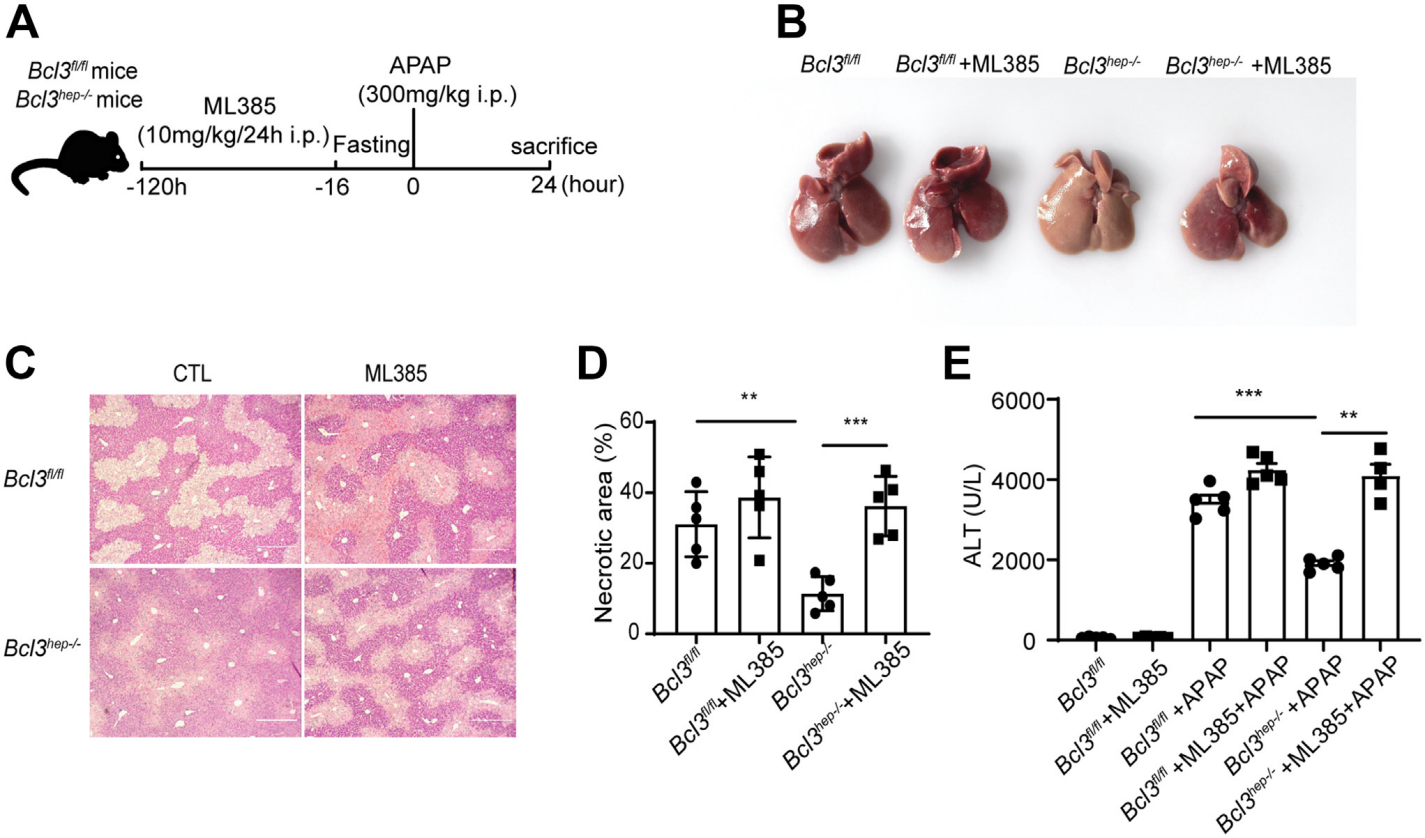
**An Nrf2 inhibitor counteract the protective effect of Bcl3 on APAP-induced liver injury.** (*A*) Experimental scheme. *Bcl3*^*fl/fl*^ and *Bcl3*^*hep-/-*^ mice were treated with the Nrf2 inhibitor ML385 (30 mg/kg/day intraperitoneally) for 5 days, fasted for 16 hours, and then administered APAP (300 mg/kg, intraperitoneally) for 24 hours before sacrifice and tissue collection. (*B*) Representative liver morphologies of mice from (*A*). (*C*) H&E staining of liver sections from mice in (*A*) (scale bar, 50 μm). (*D*) Quantification of necrotic areas in liver tissues (n = 5 per group). (*E*) Serum ALT levels (n = 5 per group). Data represent 3 independent experiments and are presented as mean ± SEM. Student *t*-test (*D, E*), ∗∗*P* < .01; ∗∗∗*P* < .001.

An Nrf2 inhibitor (ML385) used to further confirm the protective effect of Bcl3 deficiency was based on activation of the Nrf2 pathway (Figure 8*A*).^35^ As we expected, the protective effects of Bcl3 were obviously offset through ML385 administration in APAP-treated mice. Importantly, liver congestion was more serious in *Bcl3*^*hep-/-*^ mice after ML385 treatment compared with *Bcl3*^*hep-/-*^ mice without ML385 treatment (Figure 8*B*). H&E staining results further evidenced that ML385 treatment contributed to nearly liver necrosis between *Bcl3*^*hep-/-*^ mice and *Bcl3*^*fl/fl*^ mice (Figure 8*C and D*); meanwhile, the ALT level also showed the same results (Figure 8*E*). Therefore, Bcl3 deficiency plays a protective role in APAP-induced liver injury mainly by activating the Nrf2 pathway.

## Discussion

To develop novel therapeutic interventions in APAP-induced liver failure, it is essential to clearly understand the mechanisms underlying hepatocyte death. Bcl3, a critical survival factor for various cell types, is known to participate in anti-inflammatory regulation through the NF-κB pathway, as well as in cell homeostasis and metabolism.^32^ However, its role in drug-induced acute liver injury remains largely unexplored. In this study, we demonstrated that mice with liver-specific Bcl3 knockout experienced significantly less liver injury than control mice following APAP overdose. Importantly, Bcl3 deficiency did not affect the initial metabolism of APAP but significantly enhanced the biosynthesis of GSH via Nrf2 signaling activation. This study is the first to report the crucial role of Bcl3 in the antioxidant stress response, highlighting its decisive impact on hepatocyte death.

In the present study, we found that APAP overdose caused a drastic elevation in hepatic Bcl3 levels. *Bcl3*^*hep-/-*^ mice experienced significantly lower liver injury at 6 and 24 hours after APAP administration, evidenced by lower levels of ALT, AST, and necrosis areas. APAP overdose leads to the saturation of its normal metabolic clearance pathways (sulfation and glucuronidation), resulting in the formation of large amounts of NAPQI.^36^ In our study, the critical metabolic enzyme Cyp2E1 did not show significant changes with Bcl3 deficiency. The central mediator of APAP-induced acute liver injury is hepatic oxidative stress initiated by the attachment of the APAP metabolite NAPQI to mitochondrial proteins.^37^ The accumulation of ROS and peroxynitrite results in mitochondrial dysfunction, halting ATP production and causing necrosis.^3^ Rapid GSH depletion followed by its gradual replenishment is a key mechanism in APAP-induced liver injury.^38^ In the present study, the basal GSH levels were similar in both the *Bcl3*^*hep-/-*^ and Bcl3^*fl/fl*^ mice. Bcl3-deficient mice displayed notably faster GSH recovery following APAP overdose. These data showed a link between Bcl3 deficiency and GSH replenishment. GSH replenishment is regulated by the transcription factor Nrf2 that controls the expression of *Gclc* and *Gclm* genes related to the de novo synthesis of GSH.^39^ The Kelch-like ECH-associated protein 1 (Keap1)-Nrf2 axis is involved in various liver diseases including nonalcoholic fatty liver disease, alcoholic liver disease, and toxicant-induced liver injury.^40^, ^41^, ^42^, ^43^ Moreover, numerous studies on APAP-induced hepatotoxicity have implicated the involvement of Nrf2. Importantly, Nrf2 and its target proteins including GCLC and GCLM exhibited increased expression in *Bcl3*^*hep-/-*^ mice after APAP treatment. Given Bcl3’s role as a transcriptional coactivator, we investigated its potential interaction with Nrf2. Initially, co-localization of Nrf2 and Bcl3 was identified in liver tissue and hepatocytes. Immunoprecipitation experiments further confirmed a direct interaction between Bcl3 and Nrf2 in Huh7 cells, 293T cells, and liver tissue. Importantly, Bcl3 deficiency promoted Nrf2 nuclear translocation upon APAP treatment, as shown by immunofluorescence staining and cytoplasmic and nuclear protein extraction assays. Additionally, treatment with the Nrf2 inhibitor ML385 significantly compromised the protective effects observed in Bcl3-deficient conditions, indicating that the alleviation of APAP-induced liver injury by Bcl3 deficiency is Nrf2-dependent. Overall, the presence of Bcl3 inhibits Nrf2 activation, whereas Bcl3 knockout enhances Nrf2 nuclear translocation, contributing to rapid GSH replenishment and the alleviation of APAP-induced hepatocyte necrosis.

Various risk factors can induce the expression of Bcl3 leading to liver injury, such as LPS,^32^^,^^44^ high-fat diet, and alcohol.^45^ There has been some controversy regarding the role of Bcl3 in hepatocyte death in recent years, and the exact functions it plays in this process remain a subject of debate. Hu et al found that Bcl3 promotes TNF-induced hepatocyte apoptosis by regulating the deubiquitination of RIP in a CYLD-dependent manner and noted that Bcl3 does not affect FasL- or TRAIL-induced apoptosis in HepG2 or LO2 cells. Interestingly, Bcl3 played a protective role against TNF/CHX-induced apoptosis in colon cancer cells and breast cancer cells.^33^ However, Gehrke et al reported that Bcl3 protected hepatocytes from d-GalN/LPS-induced apoptosis by inhibiting caspase activation, BCL-XL degradation, and mitochondrial ROS formation. Additionally, Bcl3 overexpression alleviated Jo2-induced hepatocyte apoptosis.^32^ Although Hu’s study utilized global Bcl3 knockout mice and Gehrke’s study used hepatocyte-specific Bcl3 overexpression, the opposing functions of Bcl3 in hepatocyte apoptosis remain puzzling. Unlike TNF or FasL-induced apoptosis, APAP-induced liver injury is a classic model for studying hepatocyte necrosis.^4^^,^^46^ Although numerous studies have confirmed that inflammation and various immune cells play critical roles in the pathological progression of APAP-induced liver injury, it is clear that these events occur subsequent to the initial damage to hepatocytes. In our investigation, we utilized liver-specific Bcl3 knockout and overexpression mice to minimize the systemic effects associated with global knockout models. Our findings reveal a previously unreported mechanism in which Bcl3 deficiency protects against APAP-induced hepatocyte necrosis in a Nrf2-dependent manner. This highlights the importance of early cellular responses in mitigating the impact of APAP-induced liver injury before the onset of inflammatory and immune cell involvement.

Our results indicate that Bcl3 plays a negative role in APAP-induced oxidative stress, yet its expression is increased under these conditions. Why does the hepatocyte respond to APAP-induced oxidative stress with an up-regulation of a factor that acts to down-regulate rather than up-regulate the cellular antioxidant defenses that are needed for hepato-protection from injury? APAP overdose induces oxidative stress, leading to the production of substantial amounts of ROS. Studies have reported that oxidative stress from sources such as ultraviolet light and cellular senescence also enhances Bcl3 expression. Additionally, Bcl3 is involved in regulating inflammation and may play different roles depending on the context.^28^ For instance, Bcl3 has been shown to promote TNF-induced hepatocyte apoptosis by enhancing RIP1 deubiquitination, and TNF itself increases Bcl3 expression.^33^ However, the specific role of Bcl3 in APAP-induced inflammation remains unclear. It is possible that hepatocytes upregulate Bcl3 expression as a result of an inflammatory response; however, this hypothesis requires further investigation.

In cellular systems, antioxidant and cytoprotective responses can be categorized into 2 distinct types. The first type involves the elimination of oxidant species to mitigate cellular damage through the activation of antioxidant proteins.^47^ Under baseline conditions, Nrf2 is bound and sequestered in the cytosol by its partner protein Keap1. During oxidative stress, modifications of cysteine residues on Keap1 lead to the release of Nrf2, allowing it to translocate to the nucleus.^48^ There, Nrf2 binds to the antioxidant response element (ARE), initiating the transcription of a suite of genes critical for the antioxidant response, known as the type I antioxidant response. In our study, we found that Bcl3 knockout facilitates the nuclear translocation of Nrf2 in liver tissue and primary hepatocytes, resulting in the upregulation of antioxidant gene expression. The second type of antioxidant response involves curtailing the production of ROS. Damaged mitochondria can initiate a harmful cycle of ROS generation and subsequent cellular damage. Autophagy is currently recognized as the sole mechanism capable of efficiently removing these damaged mitochondria.^49^ Notably, APAP-induced protein adducts on cellular proteins must be cleared to preserve cellular homeostasis, and APAP administration leads to an increase in autophagosome formation, particularly in the pericentral area of the liver.^50^ Previous research from our group demonstrated that Bcl3 promotes autophagy in human T-lymphotropic virus type 1 (HTLV-1)-infected cells.^51^ However, further investigation is required to elucidate the role of Bcl3 in APAP-induced autophagy or mitophagy.

In conclusion, our study identifies a pivotal role for Bcl3 in the early stages of APAP-induced acute liver failure. We found that liver-specific Bcl3 deficiency significantly reduces hepatic oxidative stress and necrosis following APAP overdose. Mechanistically, Bcl3 interacts with Nrf2, and the absence of Bcl3 enhances the nuclear translocation of Nrf2, promoting the expression of antioxidant-related genes and facilitates GSH recovery. These findings suggest that Bcl3 is a crucial regulator of the cellular antioxidant response during APAP-induced hepatotoxicity.

## Materials and Methods

### Animal Experiments

C57BL/6N mice were purchased for Beijing Vital River Company. Liver-specific Bcl3 knockout (*Bcl3*^*hep-/-*^) mice were generated by crossing *Bcl3*^*flox/flox*^ mice with Albumin-Cre (Alb-Cre) mice in several steps. *Bcl3*^*flox/flox*^ mice were purchased from Cyagen company, and Alb-Cre mice were donated by Professor Yunwei Lou. *Bcl3*^*flox/flox*^ mice were first crossed with Alb-Cre mice, and heterozygous offspring carrying Alb-Cre transgene (*Bcl3*^*wt/flox*^-Cre) were crossed with *Bcl3*^*flox/flox*^ mice to generate homozygous mice carrying the Alb-Cre transgene (Alb-Cre-*Bcl3*^*flox/flox*^), which were used as liver-specific Bcl3 knockout (*Bcl3*^*hep-/-*^) mice, Other littermates (*Bcl3*^*fl/fl*^) carrying no Alb-Cre transgene were used as wild-type controls. All animal experiments were approved by the Animal Care and Use Committee of Xinxiang Medical University and were conducted in accordance with the Guidelines of the China Animal Welfare Legislation.

### Adeno-associated Virus 8 mediated Bcl3 overexpression

The adeno-associated virus (AAV)8-thyroxine-binding globulin (TBG)-m-Bcl3-3×flag-ZsGreen that overexpress Bcl3 in mouse liver was constructed by Hanbio Tech, with AAV8-TBG-3×flag-ZsGreen-NC serving as a control. Eight-week-old mice were injected intravenously with a single dose of virus (100 μL) containing 1.8 × 10^11^ AAV8 vector genomes for 3 weeks.

### Mouse Model of APAP-induced Liver Injury

For the APAP-induced liver injury model, 8-week-old male mice were fasted for 16 hours to deplete GSH levels and then injected intraperitoneally with a single dose of 300 mg/kg of APAP (MCE) dissolved in 60 °C phosphate-buffered saline (PBS). The mice were anesthetized via 5% chloral hydrate at various time points after APAP injection. Blood was collected at the indicated time points, and mice livers were removed and frozen in liquid nitrogen or fixed in 4% paraformaldehyde. Survival rate experiments were conducted with a lethal dose of APAP (750 mg/kg), and the time of death was recorded.

### ML385 Treatment

ML385 was sequentially dissolved in a solution containing 10% DMSO, 40% PEG300, 5% Tween 80, and 45% saline. Eight-week-old male mice were treated daily with ML385 at 30 mg/kg body weight by intraperitoneal injection, whereas control groups received the same volume of the vehicle solution. After 5 days, all mice were administered APAP to induce acute liver injury, as described previously.^52^

### Cell Line and Transfection

The Huh7 and HEK 293T cell lines were kindly provided by Professor Liangwei Duan. These cells were cultured in Dulbecco’s Modified Eagle Medium (DMEM; Gibco) supplemented with 10% fetal bovine serum (FBS; Biological Industries) and 1% penicillin-streptomycin (Beyotime). The cultures were maintained in a humidified incubator at 37 °C with 5% CO_2_. Plasmid transfections were performed using Lipo2000 reagent (Vazyme) according to the manufacturer’s protocol.

### Isolation and Stimulation of Mouse Primary Hepatocytes

Mouse primary hepatocytes (MPHs) were isolated from *Bcl3*^*fl/fl*^ and *Bcl3*^*hep-/-*^ male mice as previously described.^53^ Briefly, mice were cannulated through the portal vein, and after nicking the inferior vena cava, perfused with 10 mL of HEPES containing 5 mM EDTA followed by 15 mL of DMEM containing 0.05% collagenase Ⅱ. Livers were isolated, torn, shaken loose in hepatocyte collagenase medium, and filtered through a 70-μm cell strainer. Cells were washed at 50 × g for 2 minutes, once with culture medium, overlaid with 90% Percoll, and centrifuged at 200 × g for 10 minutes. Hepatocyte pellets were collected, washed, and counted. Hepatocytes were cultured in DMEM medium containing 10% FBS and 100 U/mL penicillin and 0.1 mg/mL streptomycin. The cells were maintained at 37 °C in a humidified incubator with 5% CO_2_ overnight and then treated with 10 μM APAP for 0, 3, or 6 hours before being harvested for analysis.

### Biochemical Analysis of Serum and Liver

ALT, AST, and LDH in serum were measured using reagent kit from Nanjing Jiancheng Bioengineering Institute; SOD and GSH in liver homogenates were measured using reagent kit from Solarbio company.

### Histology and Immunofluorescence

For H&E staining, liver tissues were fixed in 4% paraformaldehyde, embedded in paraffin, and sectioned at 5 μm. Sections were stained with H&E and examined under a light microscope (Leica). Liver sections or MPHs were incubated with primary antibodies against Nrf2 (Proteintech), GCLC (Proteintech), and Bcl3 (Santa Cruz) at 4 °C overnight. After washing 3 times with PBS, the sections were incubated with secondary antibodies for 1 hour at 37 °C. For TUNEL (Servicebio) and Mitosox (Thermofisher), stainings were performed according to the manufacturer’s instructions. Immunohistochemical sections images were obtained from microscope (Leica), and fluorescence images were captured using a confocal laser scanning microscope (Nikon).

### Western Blotting

For Western blotting, the cells or tissues were lysed in RIPA buffer (Beyotime) supplemented with a protease inhibitor cocktail (Roche Diagnostics), and lysates were centrifuged at 12,000 rpm to remove insoluble debris. Protein concentrations of the resultant lysates was determined using a bicinchoninic acid (BCA) assay kit (Beyotime). Equal amounts of proteins were boiled for 10 minutes in 5 × SDS loading buffer (Servicebio), separated by sodium dodecyl sulfate polyacrylamide gel electrophoresis (SDS-PAGE), and transferred to polyvinylidene fluoride (PVDF) membranes. The membranes were incubated in blocking buffer (5% non-fat dry milk in Tris-buffered saline with Tween 20 [TBST]) for 1 hour and then with primary antibodies at 4 °C overnight. After 3 washes with TBST, the membrane was incubated with horseradish peroxidase (HRP)-conjugated secondary antibodies (Proteintech) for 1 hour at room temperature. The membranes were washed 3 times and visualized with Luminescent image analyzer (GE). Related antibodies were listed in Table 2.

**Table 2:** Antibodies Used for Western Blot and Immunofluorescence

Antibody	Cat no.	Company
Bcl3 (Mouse)	sc-32741	Santa Cruz
Bcl3 (Rabbit)	23959-1-AP	Proteintech
Nrf2	16396-1-AP	Proteintech
GCLC	12601-1-AP	Proteintech
GCLM	14241-1-AP	Proteintech
Keap1	YT5218	Immuoway
Cyp2E1	ab28146	Abcam
β-actin	KA1035	Kemix
GAPDH	KA1037	Kemix
HRP-conjuncted goat anti-mouse	SA00001-1	Proteintech
HRP-conjugated goat anti-rabbit	SA00001-2	Proteintech
Histone H3 Polyclonal antibody	17168-1-AP	Proteintech

### Immunoprecipitation

For immunoprecipitation (IP), Huh7 cells and 293T cells transfected with Flag-Bcl3 or Nrf2 plasmid were lysed in NP40 buffer (Beyotime) and centrifuged to remove debris. The target protein was precipitated with appropriate antibodies conjugated directly to Sepharose beads (Flag M2 beads, Yeasen) or via protein A/G-conjugated Sepharose beads (Santa). Approximately 2 mg of total protein lysates were adsorbed with magnetic poles or incubated with the antibody-conjugated beads for 2 hours at room temperature or overnight at 4 °C. After the incubation, the beads were washed 5 times with NP40 buffer, and the bead-bound proteins were eluded off through boiling in denaturing SDS-gel loading buffer, followed by Western blotting analysis.

### RNA Extraction and Quantitative Real-time Polymerase Chain Reaction

Total RNA was extracted from cells or tissues with RNAiso Plus (Takara) according to the manufacturer’s protocol. The RNA concentration was determined with Nanodrop 2000 (Thermo Scientific), and 1 μg RNA was reverse transcribed using a kit (Takara). Quantitative real-time polymerase chain reaction (qRT-PCR) was performed in SYBR Green PCR Master Mix (Thermo Scientific) on 7500 FAST Real-Time PCR System (Applied Biosystems). All mRNA transcript levels were normalized to glyceraldehyde-3-phosphate dehydrogenase (GAPDH). The sequences of the primers used are listed in Table 3.

**Table 3:** Primers Used for qRT-PCR Analysis

Gene name	Sequence (5′ to 3′)
GAPDH-F	TGCAGTGGCAAAGTGGAGATT
GAPDH-R	TCGCTCCTGGAAGATGGTGAT
GCLC-F	GGCTCTCTGCACCATCACTT
GCLC-R	GTTAGAGTACCGAAGCGGGG
GCLM-F	AGGAGCTTCGGGACTGTATCC
GCLM-R	GGGACATGGTGCATTCCAAAA
Nrf2-F	CTTTAGTCAGCGACAGAAGGAC
Nrf2-R	AGGCATCTTGTTTGGGAATGTG
Cyp2e1-F	CGTTGCCTTGCTTGTCTGGA
Cyp2e1-R	AAGAAAGGAATTGGGAAAGGTCC
Bcl3-F	GCCTACTCACCCCTACTCCA
Bcl3-R	ATGTGGTGATGACAGCCAGG

qRT-PCR, quantitative real-time polymerase chain reaction.

### Quantification and Statistical Analysis

Grayscale analysis of protein bands, fluorescence intensity, and areas of necrosis in liver tissues were quantified using ImageJ (National Institutes of Health). The mean fluorescence intensity (MFI) of MitoSOX were analyzed by Flowjo_V10 (BD). Statistical significance for normally distributed data was assessed using 2-sided Student *t*-tests or 1-way analysis of variance (ANOVA) as indicated in the figure legends. For non-normally distributed data, nonparametric tests (Kruskal-Wallis with Dunn’s correction in place of ANOVA, and Mann-Whitney *U* test in place of 2-tailed *t*-test) were conducted. All statistical analyses were performed using GraphPad Prism 8 software (GraphPad Software), and a *P* value of < .05 was considered statistically significant.

## References

[1] Ghany M.G., Watkins P.B. (2023). Moving the needle to reduce acetaminophen (paracetamol) hepatotoxicity. JAMA.

[2] Jaeschke H., Ramachandran A., Chao X., Ding W.X. (2019). Emerging and established modes of cell death during acetaminophen-induced liver injury. Arch Toxicol.

[3] Yan M., Huo Y., Yin S., Hu H. (2018). Mechanisms of acetaminophen-induced liver injury and its implications for therapeutic interventions. Redox Biol.

[4] Iorga A., Dara L. (2019). Cell death in drug-induced liver injury. Adv Pharmacol.

[5] Win S., Than T.A., Min R.W. (2016). c-Jun N-terminal kinase mediates mouse liver injury through a novel Sab (SH3BP5)-dependent pathway leading to inactivation of intramitochondrial Src. Hepatology.

[6] Schattenberg J.M., Czaja M.J. (2014). Regulation of the effects of CYP2E1-induced oxidative stress by JNK signaling. Redox Biol.

[7] Seki E., Brenner D.A., Karin M. (2012). A liver full of JNK: signaling in regulation of cell function and disease pathogenesis, and clinical approaches. Gastroenterology.

[8] Sun Y., Li T.Y., Song L. (2018). Liver-specific deficiency of unc-51 like kinase 1 and 2 protects mice from acetaminophen-induced liver injury. Hepatology.

[9] Yang R., Miki K., He X. (2009). Prolonged treatment with N-acetylcystine delays liver recovery from acetaminophen hepatotoxicity. Crit Care.

[10] Palmer S., Chen Y.H. (2008). Bcl-3, a multifaceted modulator of NF-kappaB-mediated gene transcription. Immunol Res.

[11] Herrington F.D., Nibbs R.J. (2016). Regulation of the adaptive immune response by the IkappaB family protein Bcl-3. Cells.

[12] Bours V., Franzoso G., Azarenko V. (1993). The oncoprotein Bcl-3 directly transactivates through kappa B motifs via association with DNA-binding p50B homodimers. Cell.

[13] Song L., Wormann S., Ai J. (2016). BCL3 reduces the sterile inflammatory response in pancreatic and biliary tissues. Gastroenterology.

[14] Kreisel D., Sugimoto S., Tietjens J. (2011). Bcl3 prevents acute inflammatory lung injury in mice by restraining emergency granulopoiesis. J Clin Invest.

[15] Poveda J., Sanz A.B., Carrasco S. (2017). Bcl3: a regulator of NF-kappaB inducible by TWEAK in acute kidney injury with anti-inflammatory and antiapoptotic properties in tubular cells. Exp Mol Med.

[16] O’Carroll C., Moloney G., Hurley G. (2013). Bcl-3 deficiency protects against dextran-sodium sulphate-induced colitis in the mouse. Clin Exp Immunol.

[17] Gringhuis S.I., Kaptein T.M., Wevers B.A. (2014). Fucose-specific DC-SIGN signalling directs T helper cell type-2 responses via IKKepsilon- and CYLD-dependent Bcl3 activation. Nat Commun.

[18] Ruan Q., Zheng S.J., Palmer S. (2010). Roles of Bcl-3 in the pathogenesis of murine type 1 diabetes. Diabetes.

[19] Tang W., Wang H., Claudio E. (2014). The oncoprotein and transcriptional regulator Bcl-3 governs plasticity and pathogenicity of autoimmune T cells. Immunity.

[20] Zhang X., Wang H., Claudio E. (2007). A role for the IkappaB family member Bcl-3 in the control of central immunologic tolerance. Immunity.

[21] Corn R.A., Hunter C., Liou H.C. (2005). Opposing roles for RelB and Bcl-3 in regulation of T-box expressed in T cells, GATA-3, and Th effector differentiation. J Immunol.

[22] Meguro K., Suzuki K., Hosokawa J. (2015). Role of Bcl-3 in the development of follicular helper T cells and in the pathogenesis of rheumatoid arthritis. Arthritis Rheumatol.

[23] Zhang X., Paun A., Claudio E. (2013). The tumor promoter and NF-kappaB modulator Bcl-3 regulates splenic B cell development. J Immunol.

[24] Tang W., Saret S., Tian R. (2021). Bcl-3 suppresses differentiation of RORgammat(+) regulatory T cells. Immunol Cell Biol.

[25] Zhang S., Gao J., Liu S. (2021). Transcription coactivator BCL3 acts as a potential regulator of lipid metabolism through the effects on inflammation. J Inflamm Res.

[26] Liu H., Zeng L., Yang Y. (2022). Bcl-3 regulates the function of Th17 cells through raptor mediated glycolysis metabolism. Front Immunol.

[27] Liu H., Zeng L., Pan M. (2023). Bcl-3 regulates T cell function through energy metabolism. BMC Immunol.

[28] Liu H., Zeng L., Yang Y. (2022). Bcl-3: a double-edged sword in immune cells and inflammation. Front Immunol.

[29] Tu K., Liu Z., Yao B. (2016). BCL-3 promotes the tumor growth of hepatocellular carcinoma by regulating cell proliferation and the cell cycle through cyclin D1. Oncol Rep.

[30] Gehrke N., Worns M.A., Huber Y. (2016). Hepatic B cell leukemia-3 promotes hepatic steatosis and inflammation through insulin-sensitive metabolic transcription factors. J Hepatol.

[31] Shao C., Jing Y., Zhao S. (2022). LPS/Bcl3/YAP1 signaling promotes Sox9(+)HNF4alpha(+) hepatocyte-mediated liver regeneration after hepatectomy. Cell Death Dis.

[32] Gehrke N., Worns M.A., Mann A. (2022). Hepatocyte Bcl-3 protects from death-receptor mediated apoptosis and subsequent acute liver failure. Cell Death Dis.

[33] Hu Y., Zhang H., Xie N. (2022). Bcl-3 promotes TNF-induced hepatocyte apoptosis by regulating the deubiquitination of RIP1. Cell Death Differ.

[34] Jaeschke H., Ramachandran A. (2024). Acetaminophen hepatotoxicity: paradigm for understanding mechanisms of drug-induced liver injury. Annu Rev Pathol.

[35] Singh A., Venkannagari S., Oh K.H. (2016). Small molecule inhibitor of NRF2 Selectively intervenes therapeutic resistance in KEAP1-deficient NSCLC tumors. ACS Chem Biol.

[36] Stahl S.H., Yates J.W., Nicholls A.W. (2015). Systems toxicology: modelling biomarkers of glutathione homeostasis and paracetamol metabolism. Drug Discov Today Technol.

[37] Jaeschke H., McGill M.R., Ramachandran A. (2012). Oxidant stress, mitochondria, and cell death mechanisms in drug-induced liver injury: lessons learned from acetaminophen hepatotoxicity. Drug Metab Rev.

[38] Kotulkar M., Paine-Cabrera D., Abernathy S. (2023). Role of HNF4alpha-cMyc interaction in liver regeneration and recovery after acetaminophen-induced acute liver injury. Hepatology.

[39] He F., Ru X., Wen T. (2020). NRF2, a transcription factor for stress response and beyond. Int J Mol Sci.

[40] Jayasuriya R., Dhamodharan U., Ali D. (2021). Targeting Nrf2/Keap1 signaling pathway by bioactive natural agents: possible therapeutic strategy to combat liver disease. Phytomedicine.

[41] Solano-Urrusquieta A., Morales-Gonzalez J.A., Castro-Narro G.E. (2020). NRF-2 and nonalcoholic fatty liver disease. Ann Hepatol.

[42] Sun J., Fu J., Li L. (2018). Nrf2 in alcoholic liver disease. Toxicol Appl Pharmacol.

[43] Yang J.J., Tao H., Huang C., Li J. (2013). Nuclear erythroid 2-related factor 2: a novel potential therapeutic target for liver fibrosis. Food Chem Toxicol.

[44] Wessells J., Baer M., Young H.A. (2004). BCL-3 and NF-kappaB p50 attenuate lipopolysaccharide-induced inflammatory responses in macrophages. J Biol Chem.

[45] Bala S., Tang A., Catalano D. (2012). Induction of Bcl-3 by acute binge alcohol results in toll-like receptor 4/LPS tolerance. J Leukoc Biol.

[46] Jaeschke H., Adelusi O.B., Akakpo J.Y. (2021). Recommendations for the use of the acetaminophen hepatotoxicity model for mechanistic studies and how to avoid common pitfalls. Acta Pharm Sin B.

[47] Kensler T.W., Wakabayashi N., Biswal S. (2007). Cell survival responses to environmental stresses via the Keap1-Nrf2-ARE pathway. Annu Rev Pharmacol Toxicol.

[48] Chen N., Hu M., Jiang T. (2024). Insights into the molecular mechanisms, structure-activity relationships and application prospects of polysaccharides by regulating Nrf2-mediated antioxidant response. Carbohydr Polym.

[49] Ruart M., Chavarria L., Camprecios G. (2019). Impaired endothelial autophagy promotes liver fibrosis by aggravating the oxidative stress response during acute liver injury. J Hepatol.

[50] Mo R., Lai R., Lu J. (2018). Enhanced autophagy contributes to protective effects of IL-22 against acetaminophen-induced liver injury. Theranostics.

[51] Wang J., Li J., Huang Y. (2013). Bcl-3 suppresses Tax-induced NF-kappaB activation through p65 nuclear translocation blockage in HTLV-1-infected cells. Int J Oncol.

[52] Li S., Zhuge A., Xia J. (2023). Bifidobacterium longum R0175 protects mice against APAP-induced liver injury by modulating the Nrf2 pathway. Free Radic Biol Med.

[53] Charni-Natan M., Goldstein I. (2020). Protocol for primary mouse hepatocyte isolation. STAR Protoc.

